# Forced eye closure-induced reflex seizure and non-ketotic hyperglycemia

**DOI:** 10.4103/0256-4947.55319

**Published:** 2009

**Authors:** Raziye Tiras, Aytul Mutlu, Serkan Ozben, Tuba Aydemir, Feriha Ozer

**Affiliations:** a Department of Neurology, Haseki Educational and Research Hospital, Istanbul, Turkey; b Department of Neurology and Movement Disorders Outpatient Clinic, Istanbul, Turkey

## Abstract

We report an uncommon case of 53-year-old female patient with partial seizure induced by forced voluntary eye closure due to non-ketotic hyperglycemia. The initial laboratory tests showed an elevated blood glucose level of 550 mg/dL but no evidence of ketosis. Brain magnetic resonance imaging was normal. When the blood glucose levels decreased slowly to about 150 mg/dL in five days, the seizures ended completely. No anticonvulsants were used. Since seizures are generally refractory to antiepileptic medication, control of blood glucose is essential.

Reflex epilepsy is a rare form of epilepsy that develops as a result of certain specific stimulations. The most frequent form of reflex epileptic seizures occur with visual stimuli.^1^–^3^ Reflex seizures induced by non-visual stimuli are rarely reported. Seizures may also be evoked by other activities such as decision making, thinking, calculations, reading and writing, eating, listening to music, proprioception and hot water.^4^ We report an uncommon case of a middle-aged diabetic patients presenting with uncontrolled diabetes and partial seizures induced by forced voluntary eye closure.

## CASE

**Figure 1:**
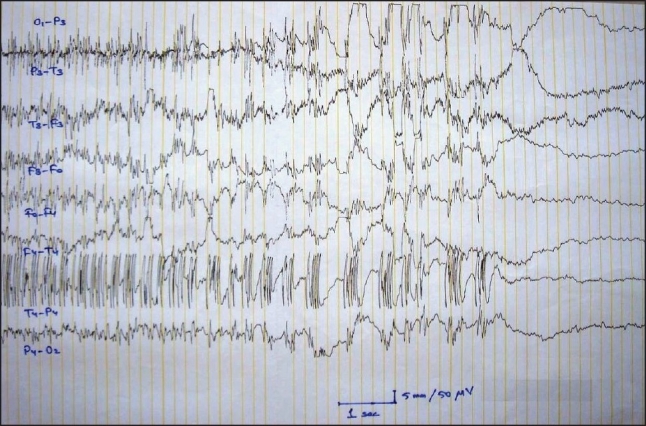
Discharges of high amplitude spike-waves and multiple spikes-waves which localized to the right temporoparietal electrodes and involved adjacent areas.

A 53-year-old female with poorly controlled diabetes mellitus (DM) was brought to the emergency department with focal motor clonic seizures induced by forced voluntary eye closure due to rubbing her eyes. During hospitalization it was observed that seizures began when she closed her eyes strongly. Every time she repeated the eye closure the seizures recurred. The seizures started two days previously and occurred with each forced voluntary eye closure. She had no seizure with spontaneous eye blinking or sleeping, or with horizontal, vertical or rotatory eye movements. The seizures occured in the left orbicularis oculi, and then spread to the ipsilateral angularis oris and upper extremity, turning to the left side of the head. They lasted 2 to 3 minutes. The patient did not lose consciousness during seizures. She had had type 2 DM for eight years and her past medical history revealed no other significant disease. She had no history of seizures or other neurological disorder. She was using only insulin as her medication. The physical examination, including the neurological examination of the patient, revealed no abnormality. Her visual acuity, pupil reactions to light and eye movements were normal. The laboratory tests showed an elevated blood glucose level of 550 mg/dL but no evidence of ketosis. Serum osmolarity was 279.8 mOsm/L. Her ictal electroencephalogram (EEG) showed discharges of high amplitude spike-waves and multiple spikes-waves which localized to the right temporoparietal electrodes involving to the adjacent area. (Figure 1). The photoparoxysmal response was not observed during the photic stimulation. Brain magnetic resonance imaging (MRI) was normal. Functional MR and single photon emission computed tomography (SPECT) were not performed. Seizure control was achieved immediately after the glucose level fell to normal levels. When the blood glucose levels decreased slowly to about 150 mg/dL in five days, the seizures ended completely. No anticonvulsants were used. During the next 3 months follow-up, no seizures occured. The patient had been free of seizures with effective control of blood glucose.

## DISCUSSION

Seizures are a rare but well recognized manifestation of severe hyperglycemia, particularly in non-ketotic hyperglycemic state (NKH). Focal epileptic seizure may be induced by non-ketotic hyperosmolar diabetic comas accompanied by severe hyperglycemia, hyperosmolality and dehydration with minimal or no ketoacidosis.^5^ In this condition, focal motor seizures and epilepsy partialis continua (EPC) are the most common type, although generalized tonic-clonic seizures may also occur. Focal seizures have been reported in 19% of patients with NKH6 and focal seizures induced by eye movements have also been reported with NKH.^7^

The pathogenesis of focal seizures in NKH has not been completely understood. While focal seizures associated with NKH are refractory to antiepileptic drugs, they may be treated with adequate control of blood glucose. However, hyperglycemia alone is not an explanatory cause of seizures.^6^,^8^,^9^ One of the suggested mechanisms is presence of a hyperosmolar gradient due to hyperglycemia between intracellular and extracellular neuronal compartments with subsequent dehydration, which may produce partial seizures.^10^ The Krebs cycle is also inhibited in hyperglicemia.^11^,^12^ This may cause an increase in gamma-aminobutyric acid (GABA) metabolism in the succinil semialdehyde pathway. Depressed brain GABA levels decreases the seizure threshold, decreasing the level of GABA, which is known to be an inhibitory neurotransmitter in the central nervous system. Due to depressed brain GABA levels, the seizure threshold decreases.

Reflex epilepsy refers to seizures that are regularly precipitated by a specific identifiable stimulus.^2^ Based on the provocation mechanism, “reflex” seizure can be divided into a simple and a complex group.^13^ Triggering factors include visual, auditory, somatosensory, mental, motor and others.^2^,^4^,^14^ In the presence of a cortical lesion, hyperglycemia can facilitate an increase in frequency of focal motor seizures.^7^,^15^ Localized tissue hypoxia may account for focal seizure activity in diabetics.^15^ On the other hand, in cases with NKH, focal motor seizures related to posture have been reported, and it was suggested that proprioceptive-induced reflex seizures were related to unstable levels of blood glucose.^11^,^15^,^16^ In these cases the seizures can result from active or passive movements of a limb, with or without any brain lesion.^11^,^15^,^16^ In our case, there was no lesion on MRI.

Various abnormalities in ictal EEG were reported in seizures with NKH. In some cases ictal EEG demonstrated periodic lateralized epileptiform discharges correlated with clonic contractions. In some, focal seizure discharge was recorded in the frontal, parieto-occipital or temporal region, and in the others no paroxysmal activity was recorded.^4^,^6^,^17^ In our patient, ictal EEG showed had discharges of high amplituded spike-waves and multiple spikes-waves which localized to the right temporoparietal electrodes involving the adjacent area. Photoparoxysmal response was not observed during the photic stimulation.

Eye closure and closed-eyes tend to increase spontaneous EEG paroxysmal abnormalities. Previously, the effect of darkness and proprioceptive impulses on EEG abnormalities, which occur after closing of the eyes and disappear when the eyes are opened, were reported.^18^ It was hypothesised that proprioceptive impulses generated by eye closure or the mechanisms associated with moving the eyes may trigger paroxysmal abnormalities.^18^ In our case, a partial motor seizure was occurring at the eye circle during forced voluntary eye closure at the base of NKH. It may be related to increased GABA metabolism correlated to hyperglycemia without any ischemia. It was suggested that increased activity in reflex pathways might be secondary to the possibility of decreased GABA utilization in cortical and subcortical levels.^11^,^19^ Seizures induced by forced eye closure are a rare but well-recognized form of reflex epilepsy. In our patient, these reflex focal seizures might be triggered by proprioceptive stimuli due to the pressure during strong closing of her eyes. Our patient had no photic sensitivity and, visual symptoms were not revealed by the patient during the seizure. Seizure control was achieved after she became normoglycemic and with adequate control of blood glucose, she continued to remain seizure free even by doing the movement, which previously induced the seizure. It is essential to improve metabolic deficiency for seizure control because seizures are generally refractory to anti-epileptic drugs and patients become seizure free with improvement of metabolic deficiency.
